# Use of the 95-degree angled blade plate with biological and mechanical augmentation to treat proximal femur non-unions: a case series

**DOI:** 10.1186/s12891-022-05089-z

**Published:** 2022-02-28

**Authors:** Giovanni Vicenti, Giuseppe Solarino, Davide Bizzoca, Filippo Simone, Giuseppe Maccagnano, Giacomo Zavattini, Guglielmo Ottaviani, Massimiliano Carrozzo, Claudio Buono, Domenico Zaccari, Biagio Moretti

**Affiliations:** 1 Department of Basic Medical Sciences, Neuroscience and Sense Organs, School of Medicine, University of Bari “Aldo Moro”, AOU Consorziale Policlinico, Orthopaedic & Trauma Unit, Bari, Italy; 2 University of Bari “Aldo Moro, PhD course in Public Health, Clinical Medicine, and Oncology, Piazza Giulio Cesare 11, 70100 Bari, Italy; 3 Riuniti Hospital, Foggia, Italy

**Keywords:** femoral non-unions, biologic augmentation, mechanical augmentation, proximal femur fractures, subtrochanteric fractures, osteoporosis, bone fragility, reaming irrigation aspiration (ria)

## Abstract

**Background:**

Intertrochanteric and subtrochanteric non-union are rare but challenging complications. In the present study, we investigate the use of a 95° blade, in association with biological and mechanical augmentation, in the management of intertrochanteric and subtrochanteric femoral non-unions.

**Methods:**

Between October 2015 and February 2021, a retrospective cohort study was conducted at our institution to investigate the use of a 95° blade, in association with biological and mechanical augmentation, in the management of intertrochanteric and subtrochanteric femoral non-unions, following the mechanical failure of the first device. All the patients underwent a clinical and radiographic follow-up at 6 weeks, 3, 6, 9, 12 and 18 months; at each follow-up, a plain radiograph of the femur was performed and patients were assessed using Harris Hip Score (HHS) and the Short Form-12 (SF-12) questionnaire.

**Results:**

From October 2015 and February 2021, 40 proximal femur non-unions were managed at our Institution. Fifteen patients out of forty (37.5%) met the inclusion criteria. The main data of the study are summarized in Table 1; patients’ mean was 57 years old (range 19–83); 10 males and 5 females were included in the study. All the patients completely healed clinically and radiologically at an average of 6.1 months (range 4–13). All these patients returned to their pre-injury mobility status. During an average follow-up period of 25 months (range 8–60), the observed complications included wound dehiscence, which was treated with a superficial surgical debridement, a below-the-knee deep vein thrombosis, and a blade plate failure 3 months after the first revision surgery.

**Conclusions:**

This study shows the treatment of inter-and sub-trochanteric non-unions with a 95° blade plate, medial strut allograft, and bone autograft obtained with RIA system, together with a varus malalignment correction, leads to a high percentage of bone healing, with a low incidence of complications and good clinical outcome.

## Introduction

Hip fractures are the most treated fractures around the world [1]. Out of these, intertrochanteric fractures account for almost 50% of these injuries, while subtrochanteric fractures account for approximately 7 to 34% and have a bimodal distribution [2–6]. If young patients are generally involved in high-energy traumatic events (e.g. motor vehicle collisions), elderly patients usually undergo low-energy traumas [7].

Additionally, in the last recent years, a relevant increase in the incidence of subtrochanteric fractures has been detected, partially due to the use of bisphosphonates, especially alendronate, which could promote the genesis of atypical proximal femoral fractures, by weakening the femur lateral cortex [8]. The subtrochanteric area has peculiar features since, from an anatomic point of view, it is an area with a cortical bone predominance, linked to a very poor vascularization, which may cause a difficult and delayed bone healing process after a fracture [9]. Moreover, the peak stress in this segment could reach 57 kPa, i.e. the highest value in the entire human skeleton; moreover, compression forces are prevalent on the medial side, whereas shear forces prevail on the lateral side [10–13]. This biomechanical aspect leads to many relevant problems, such as implant fatigue and fixation failure, if the fracture does not promptly heal. Besides that, the anatomical features of the subtrochanteric region, with the deforming strain of abduction from the gluteus medius, flexion and external rotation from the iliopsoas, adduction and shortening of the shaft from the hamstrings and adductors, as well as the degree of comminution of the medial cortical buttress at the level of the fracture constitute a surgical challenge for the orthopaedic surgeon [14]. Thus, the incidence of non-unions or delayed unions in these fractures varies from 7 to 20% [15–17].

On the other hand, trochanteric fractures, thanks to a better bone vascularization of this anatomical region and the smaller deforming moments here acting, are considerably less frequently involved in the above-mentioned complications. However, in selected cases, including fractures with posteromedial wall comminution, severe osteoporotic bone, inaccurate fracture reduction and poor endomedullary nail implantation, a non-union incidence of 1–2% is reported [18–21].

In the treatment of intertrochanteric femoral fractures, two different surgical implants are commonly used: intramedullary nail (long Recon or straight) and extramedullary devices. Intramedullary fixation devices are favoured over the extra-medullary fixation, due to the shorter lever arm, the less bending movement across the fracture site and a better load sharing through the implant [22–26]. For all these reasons, the intramedullary nail fixation rate for trochanteric fractures rose from 3 to 67% over the last two decades. However, the use of this device has relatively stopped the incidence of non-union or delayed union in the subtrochanteric region, which varies from 7 to 20% [16]. The main reason for non-union in intramedullary fixation is the soft tissue interposing, a complication that requires the fracture open reduction [27]. The management of such a kind of injury has always represented a dilemma for orthopaedic surgeons during the last decades. The main variables that could influence the surgical treatment include bone quality, patient’s age and hip osteoarthritis; in presence of good bone stock and young age, reintervention with internal fixation is the treatment of choice [9]. It could be performed with a great variety of implants, such as 95°-130° angled blade plates, with or without the valgus intertrochanteric osteotomy (VITO) or hip screw and side plate (SHS), with or without bone grafting and cementation, to regain the limb’s length and rotation [28].

In the present study, we investigate the use of a 95° blade, in association with biological and mechanical augmentation, in the management of intertrochanteric and subtrochanteric femoral non-unions.

## Methods

Between October 2015 and February 2021, a retrospective cohort study was conducted at our institution to investigate the use of a 95° blade, in association with biological and mechanical augmentation, in the management of intertrochanteric and subtrochanteric femoral non-unions, following the mechanical failure of the first device. Ethical clearance was obtained from the Local Ethical Committee (Prot. n. 5556/2018), as per the 1964 Declaration of Helsinki, and all the patients gave informed consent before enrollment in the study.

Revision surgical procedure was performed by the same orthopaedic surgeon at the Traumatology Department of our Hospital. Non-union was defined as the absence of radiographic healing progression 6 months after surgery, or hardware failure at more than 5 months after surgery. All the atrophic aseptic inter- and subtrochanteric non-unions with mechanical failure presented to our institution were included in this study. The exclusion criteria were hypertrophic non-unions, pathologic fractures, signs and symptoms of infection (i.e., increased CRP or ESR), and subtrochanteric atypical fractures. This was a monocentric, single-surgeon study.

The following data were collected: patients’ demographics, initial fracture pattern, first surgical procedure, time from primary surgery to reintervention, revision surgery complications and time to final union. Plain radiographs of the pelvis, hip and femur, and a CT scan of the affected femur were performed in all the patients before the surgical revision procedure.

**Fig. 1:**
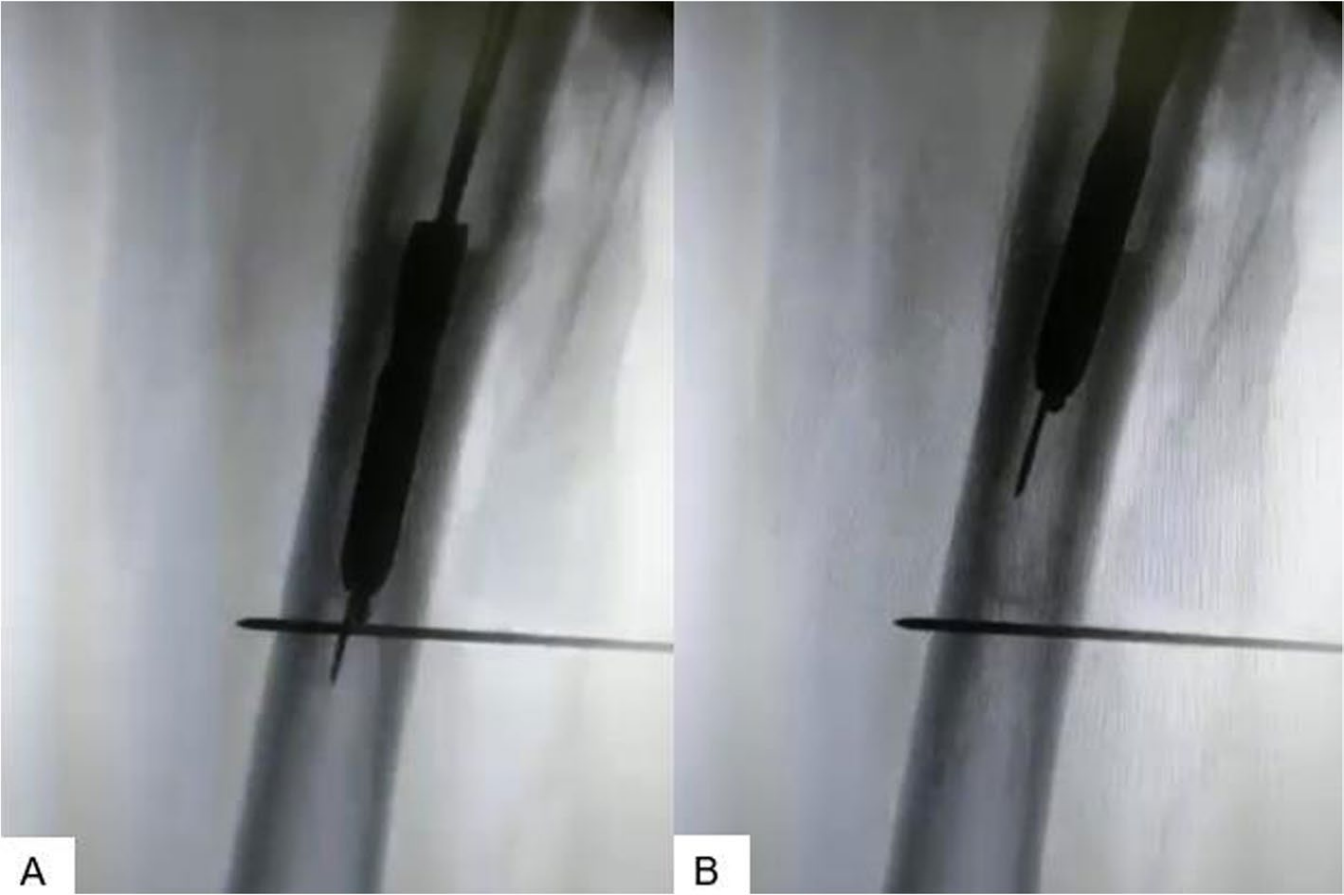
(**a-b**)- “2 k wires extracting technique”. A smart technique in all the cases in which the extractor cannot be used. A k wire is straight, and it is used to add width only, while the other one has got a curve ending to pass through the nail easily but pulling it out the opposite way. The perpendicular K wire avoids the distal migration of the nail

The surgical procedures were performed by the same surgical and anesthesiology team, under spinal anaesthesia. After positioning the patient supine on a traction table, the Reaming Irrigation Aspiration (RIA) system (De Puy Synthes, Raynham, Massachusetts, USA) was used in the contralateral femur to obtain the bone autograft. The broken intramedullary device was then removed from the non-union site, using a universal extraction system. In three cases out of 15, this extractor could not be used, therefore the “two k-wires technique” (Fig. 1) was used to remove the nail. Then soft tissue debridement and non-union site cruentation were performed. Deep samples were also taken sent to exclude the presence of a septic non-union. The patients were then treated with a 95° blade plate (DePuy Synthes), a medial femoral strut allograft, and the bone autograft taken from the contralateral femur. The fractures were reduced with the use of a screw jack to give compression and decrease the varus malalignment. In 6 patients, with an oblique fracture line, a lag screw was used. In 2 cases used Bioactive glass (FIBERGRAFT®, DePuy Synthes, North America Inc., West Chester, PA, USA). In no case, we have performed a valgus osteotomy (VITO or VSTO). Finally, the bone autograft was implanted at the debrided non-union site and a layered suture was performed.

The postoperative protocol included partial weight bearing using two crutches for 4–6 weeks, followed by a progressive increase to full weight-bearing to the patient’s tolerance. Thromboprophylaxis with Low Molecular Weight Heparin (LMWH) was administered for 4–6 weeks. All the patients underwent a clinical and radiographic follow-up at 6 weeks, 3, 6, 9, 12 and 18 months; at each follow-up, a plain radiograph of the femur was performed and patients were assessed using Harris Hip Score (HHS) and the Short Form-12 (SF-12) questionnaire.

## Results

From October 2015 and February 2021, 40 proximal femur non-unions were managed at our Institution. Fifteen patients out of forty (37.5%) met the inclusion criteria; the reaming patients were excluded for the following reasons: lost to follow-up (*n* = 5); subtrochanteric atypical fractures (*n* = 17); infected non-union (*n* = 3).

The main data of the study are summarized in Table 1; patients’ mean age was 57 years old (range 19–83); 10 males and 5 females were included in the study.

**Table 1:** Main data of the study

Pt	Age/gender	Type of trauma	Fracture classification	Time to failure (months)	Union (months)	Complications
1	19/male	High	Subtrochanteric	5	7	N/A
2	54/male	High	Subtrochanteric	4	5	Wound dehiscence
3	72/male	Low	Subtrochanteric	7	7	N/A
4	22/Female	High	Subtrochanteric	6	4	N/A
5	55/male	High	Subtrochanteric	5	4	N/A
6	83/female	Low	Intertrochanteric	3	8	Below knee deep vein thrombosis
7	41/male	High	Subtrochanteric	9	13	Breakage of blade plate-second revision to double plate construct
8	63/female	Low	Intertrochanteric	7	6	N/A
9	49/male	High	Subtrochanteric	5	4	N/A
10	58/female	High	Intertrochanteric	5	5	N/A
11	74/male	Low	Intertrochanteric	6	7	N/A
12	68/male	high	Subtrochanteric	5	3	N/A
13	70/female	Low	Subtrochanteric	6	7	N/A
14	58/male	High	Subtrochanteric	4	5	N/A
15	64/male	High	Subtrochanteric	5	7	N/A

All the primary surgical procedures failed because of mechanical problems: in 10 out of 14 IMN cases, the nail broke in the metaphyseal part, as described by Giannoudis et al. [9]. This is a critical area, where the forces are transmitted from the femoral neck to the diaphysis, and the cross-sectional area of the nail is reduced by approximately 70%. The average time from the first surgery to the device failure was 5 months (range 3–9 months). In the remaining 4 cases, the nail was intact, but the fracture non-union was evident. In the only non-union case managed with an SHS, this device broke just above the first cortical screw. In all the cases, the non-union was maligned in varus.

**Fig. 2:**
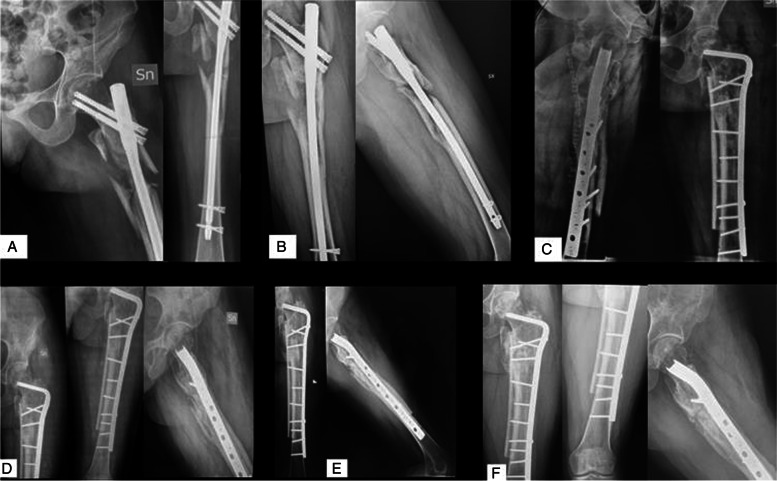
**A** AP and LL radiograph of femur subtrochanteric fracture of a 22 years old girl, due to a high energy trauma; **B** AP and LL radiographs at 6 months follow-up, showing a hypertrophic non-union; **C** AP and LL of the reintervention with blade plate, medial strut allograft and blade plate failure after 3 months; **D** AP and LL radiograph of postoperative at 2 months follow-up; **E** AP and LL radiograph at 4 months follow-up; f) AP and LL radiograph at 9 months follow-up

**Fig. 3:**
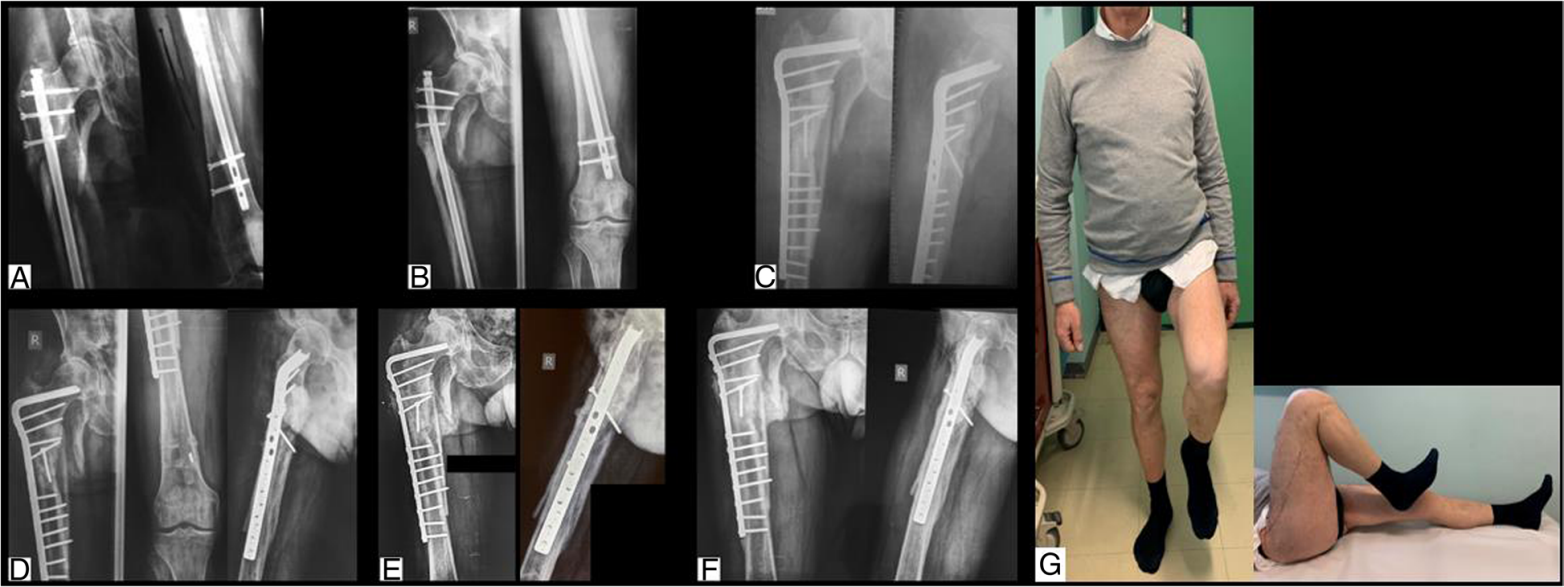
**A** radiograph of femur subtrochanteric fracture of a 55 years old man, following a high-energy trauma; **B** radiographs of the non-union with a broken EM nail, at 5 months post-op; **C** AP and LL radiographs of the reintervention with 95° blade plate, medial strut allograft, RIA system and lag screw; **D** AP and LL radiographs at 2 months follow-up; **E** AP and LL radiograph at 4 months follow-up; **F** AP and LL radiograph at 9 months follow-up; **G** clinical evaluation of the patient at 9 months follow-up

**Fig. 4:**
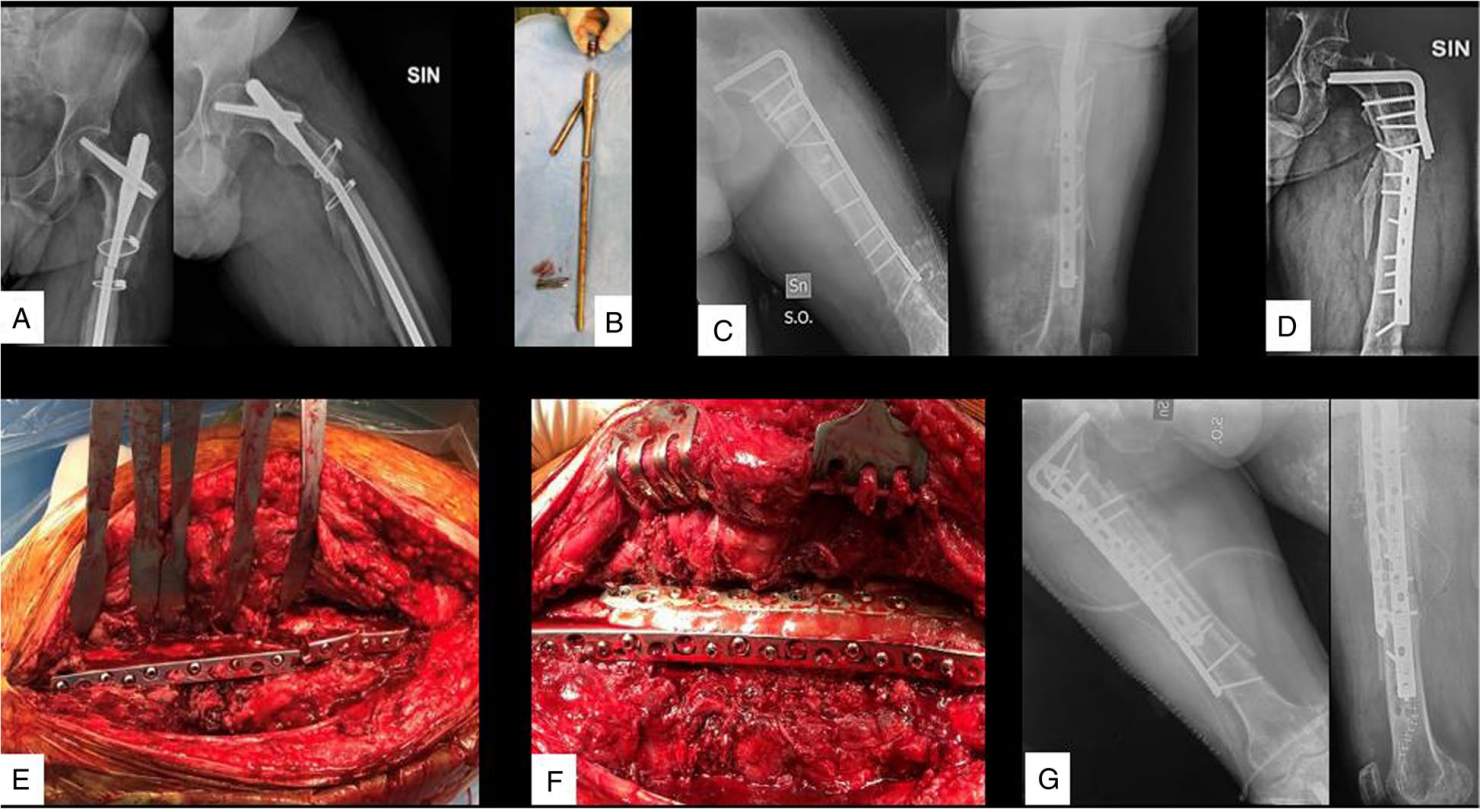
**A** AP and LL radiographs of the femur, showing a subtrochanteric non-union with a broken EM nail, at 1 year after the primary surgery; **B** intra-operative image of the broken EM nail; **C** AP and LL radiographs of the first reintervention with 95°blade plate, medial strut allograft and RIA autograft; **D** blade plate failure, at 3 months follow-up; **E** intraoperative image of the broken blade plate; **F** intraoperative image with the double-plating revision technique and medial strut allograft; **G** AP and LL radiographs of the second reintervention

All the patients completely healed clinically and radiologically at an average of 6.1 months (range 4–13) (Figs. 2 and 3). All these patients returned to their pre-injury mobility status. During an average follow-up period of 25 months (range 8–60), the observed complications included wound dehiscence, treated with a superficial surgical debridement, a below-the-knee deep vein thrombosis, and a blade plate failure 3 months after the first revision surgery. This case had second revision surgery with a double-plate construct (95° blade plate and an anterior femoral plate), with a medial femoral strut allograft and RIA graft. The bone union was reached after 7 months (Fig. 4).

The mean HHS gradually increased in all the patients, starting from 50.1 (at 3 months follow-up), reaching 72.3 at 6 months follow-up, 76.5 at 9 months, 82.4 at 12 months and getting finally to 86.7 to the last control (18 months).

The SF-12 score, on the contrary, increased slowly during the first months, due to the residual pain of the surgery and the initial limitations to the movement, but at the end reached a high peak, just as the HHS score. The mean scores were: 45.4 at 3 months, 55.6 at 6 months, 69.4 at 9 months, 78.4 at 12 months, ending with 85.3 at the 18th-month follow-up.

## Discussions

Mechanical failure of trochanteric and subtrochanteric fractures is an emerging complication due to the increasing prevalence of proximal femur fragility fractures [2, 3]. Trochanteric and subtrochanteric non-unions are caused by several factors, including both surgeon’s related (i.e., poor surgical technique; implant choice; fracture site exploration; lack of a post-operative osteoporotic therapy) and patients related factors (i.e., comorbidities, postoperative complications liable to delate the rehabilitation timing; severe osteoporosis).

The treatment of a trochanteric and subtrochanteric non-union is a highly challenging endeavour for orthopaedic surgeons and requires a sound healing and rehabilitative process. The implant choice plays a central role in the management of such a complex injury. Currently, 289 cases of inter- and sub-trochanteric non-unions have been described in the literature by 11 different authors (Table 2). 190 cases out of 289 (65.7%) were treated with a blade plate and the 95° blade plate was employed in 117 cases out of 190 cases (61.57%). Thus, this device is the most used in revision surgery for inter- and sub-trochanteric non-unions.

**Table 2:** Reintervention for inter- and sub-trochanteric femoral non-unions

Authors	Year	N° of patients	Mean age, yrs	Fixation	Bone graft	Union rate and mean time to union, mths	Outcome	Mean follow-up, mths
Intertrochanteric femoral non-unions								
Haidukewych [29]	2003	20	58	Blade plate(11), DHS (5), DCS (3), Cephalomedullary nail (1)	Autograft (17), allograft (3)	95% (19/20) TTU = n/a	16/19 w/o pain 0 AVN	27
Said [30]	2006	26	61	DHS (8), DHS + VSTO (6), blade plate + VSTO (4), THA (3), endoprosthesis (5)	None	100% (18/18) TTU = 4	16/18 w/o pain, 2/18 occasional pain 1 AVN	31
Xue [31]	2017	23	60	DHS(3), Cephalomedullary nail (9) proximal locking plate (3) THA (4) antibiotic rod staged to proximal locking plate (4)	Iliac crest autograft (6) None (13)	100% (19/19) TTU = 4,7	HHS: 28,9 (pre-op) to 83,8 (post-op)	16
Lawrenz [32]	2019	2	64	95° blade plate (2)	None (2)	100% (2/2) TTU = n/a	**First revision** 95° blade plate (1) 130° blade plate (1) **Second revision** 130° blade plate (1) 2/2 no pain at 12 months post-op FU	36
Subtrochanteric femoral non-unions								
Haidukewych [33]	2004	21	55	Cephalomedullary nail (8) Standard anterograde IMN (7) 95° blade plate (5) DHS (1) 95° DCS (1) dual large-fragment plates (1)	Autograft (8) Allograft (6) Both (3) Free vascularized fibula (1)	95% (20/21) TTU = n/a	16/20 with no pain 4/20 mild pain	12
Barquet [34]	2004	26	63	Long gamma cephalomedullary nail	Autograft (5) None (21)	96% (25/26) TTU = 7	21/26 restored to preinjury functional status	27
De vries [17]	2006	33	57	95° blade plate (24) 100–125° blade plate (7) 90°blade plate (2)	Iliac crest autograft (13) Local bone graft (1) DBX (10)	97% (32/33) TTU = 5	25/33 good or excellent Merle d’Aubigne score 5 complications requiring reoperation	31
Park [35]	2011	16	58	Blade plate (16)	Iliac crest autograft (1/14)	94% (15/16) TTU = 7	**Revision:** THA for AVN and mechanical failure (1) 1/14 great trochanter bursitis 12/14 no pain HHS: 88 (12 months post-op) Sanders functional rating scale: 14/16 (88%) good or excellent	24
Giannoudis [13]	2013	14	65	95° blade plate (11) IMN (3)	RIA graft (14) Osteoinductive factor (Osigraft Olympus) (14) MSCs from the iliac crest (14)	93% (13/14) TTU = 7	8/14 no complications 2/14 died during follow up 1/14 revision to double plate construct 1/14 pulmonary embolism 1/14 below knee deep vein thrombosis 16 failures, of which 11 were from the infected group	26
Amorosa [36]	2014	71 (57 non-infected non-unions, 21 infected non-unions)	60	95° blade plate (71)	n/a	91,2% in the not infected group TTU = 6		24
Rollo [27]	2016	35	72,4	blade plate (35)	Femoral medial strut allograft (22) None (13)	100% (22/22) in the strut allograft group TTU = 8 69% (9/13) in no allograft group TTU = 10	HHS for allograft group: 18.3 (pre-op) 85.3 (post-op) HHS for no allograft group 17.9 (pre-op) 83.2 (post-op)	12
De Biase [37]	2018	2	72	Blade plate (2)	None (2)	100% (2/2) TTU = 6	n/a	36

In the present study, the treatment of 15 proximal femur non-unions (mean age: 57 years old; male:10, female:5) managed with a 95-degree angled blade plate, in association with biological and mechanical augmentation. All the patients included in the present study completely healed clinically and radiologically at an average of 6.1 months (range 4–13). All these patients returned to their pre-injury mobility status. During an average follow-up period of 25 months (range 8–60), the observed complications included wound dehiscence, which was treated with a superficial surgical debridement, a below-the-knee deep vein thrombosis, and a blade plate failure 3 months after the first revision surgery. The findings of the present study show the 95-degree angled plates are a valid device in the management of proximal femur non-unions.

Wu et al. reported an exchange nailing to treat aseptic non-unions of the trochanteric region with excellent results, recommending an over-reaming of 1 mm or more [38]. Charnley and Ward [39] successfully treated two non-unions with a reconstruction nail and suggested its use, especially in the elderly, since it may allow an earlier full weight-bearing, compared to a blade plate. Haidukewych and Berry published the results of the treatment of 23 subtrochanteric non-unions with both intra- and extramedullary implants (15 versus 8, respectively) [33]. In 20 patients out of 21 patients available for follow-up, the union was reached. Barquet et al. reported the results of the treatment of 29 patients with a non-infected subtrochanteric non-union with a long gamma nail [34]. In two patients, hardware failure necessitated re-intervention with a long gamma nail to reach union. Finally, 25 of the 26 non-unions healed.

All these papers clearly show that intramedullary reintervention is a suitable treatment for these fractures. The only contraindication for its use is a malalignment associated with a leak of compression on the bone fragments, both problems that can be barely corrected by a second nail; in this case, the blade plate represents a good compromise.

The varus malalignment is a well-recognized risk factor for failure and non-union of these fractures [31], which usually works together with an increased bending stress at the medial femoral cortex and comminution of the medial buttress [40]. For all these reasons, treating varus malalignment represent the priority in a reintervention, and a valgus intertrochanteric osteotomy (VITO) could represent a valid solution. As reported by Muller et al., this technique is used in case of a head shaft angle (HAS) ≤ 90°, followed by a 110°/120° angled blade plate to restore the anatomy and length of the limb. In a recent work published by Bhowmich et al. [18], it is proposed an algorithm for decision making in the management of these injuries based on fracture pattern, anatomy, the status of the bone union and quality of the bone.

Many papers in the last decades have shown great results with the blade plate reintervention, both in intertrochanteric and subtrochanteric revisions (Table 1). Haidukewych et al. [29] reported the results of 11 intertrochanteric non-unions managed with blade plate and autograft. These authors observed r a bone union rate of 95%; none of the patients showed femoral head avascular necrosis at 27 months follow-up [29]. Said et al. [30] treated 4 subtrochanteric non-unions with blade plate and valgus subtrochanteric osteotomy (VSTO) and no bone graft, reaching 100% of bone union at a mean time of 4 months. Lawrenz et al. [32] performed blade plate reintervention on 2 patients, both followed by a revision with a 95° blade plate and a 130° blade plate, the latter together with a VITO. The first one, on the contrary, had a second revision with a 130° blade plate and VITO, within a few months after surgery, because of a mechanical failure. Both patients presented no pain at a 36-month follow-up. Haidukewych et al. [33] described the treatment of 5 patients with a 95° blade plate, together with an autograft, reporting bone union in all the patients, and residual mild pain in just one out of 5 at 12 months follow-up. De Vries et al. [17] treated 33 patients with subtrochanteric non-union with a 95° blade plate in 24 cases, a 125° blade plate in 7 cases and a 90° blade plate in 2 cases. In 13 cases these authors used an iliac crest autograft, a DBX Putty in 10 cases, and one case a local bone graft. The bone union was 97% at a mean follow-up of 5 months, with good or excellent results at Merle d’Aubigne score at a 31-month follow-up.

Park et al. [35] described treatment with blade plate for 16 subtrochanteric non-union, in one case together with an iliac crest autograft. Bone union was reached in 94% of patients at a mean time of 7 months. In one case, there was a mechanical failure with AVN that required a THA, while in another case there was great trochanter bursitis. HHS score was 88 at a 24-month postoperative follow-up, and Sanders functional rating scale was good or excellent in 88% of patients.

Giannoudis et al. [9] treated 11 patients with subtrochanteric non-union with a 95° blade plate, together with contralateral RIA® graft, osteoinductive factors (Osigraft®Olympus) and MSCs from the iliac crest, pursuing the so-called “Diamond concept”. In 13 cases out of 14 (93%), there was a bone union at a mean time of 7 months, although in a case it was necessary a revision to a double plate construct.

Rollo et al. [27] treated 35 subtrochanteric non-unions with a blade plate, in 22 cases together with a femoral medial strut allograft, while in the remaining 13 cases no allograft was used. In the first group, the bone union was reached in all cases at a mean time of 8 months, while in the second group 4 patients had a mechanical failure, which required a reintervention with a double plate construct. The mean time of bone union was 8 months in the first group and 10 months in the second group. HHS score improved in the first group from 18.3 (pre-op) to 85.3 (12 months post-op), while in the second group from 17.9 (pre-op) to 83.2 (12 months post-op). De Biase et al. [37] reported the treatment of 2 patients with subtrochanteric non-unions treated with blade plate. Both the patients reached bone union at a mean time of 6 months. Amorosa et al. [36] described the use of a 95° blade plate to 71 patients with subtrochanteric or distal femur non-union. The overall rate of healing was 77.5%, but more specifically was 91.2% for non-infected non-unions and 47.6% for infected non-unions. They concluded this device is a very effective reduction aid for the aseptic non-unions of the proximal and distal femur with acceptable healing rates.

In the last few years, a great debate has been reserved for bone grafting in trochanteric revision surgery. In a recent article by Mardani-Kivi et al. [41], 41 patients with subtrochanteric non-unions were treated with autogenous bridging bone grafting (corticocancellous bone harvested from the iliac crest) and double-plate fixation. An infected non-union was observed in 8 patients out of 41 [37]. The full union was obtained even in the infected cases, with only a case of deep vein thrombosis and a case of pulmonary embolism. In a similar study, Odeh et al. [42] reported the outcomes of rigid internal fixation with autogenous bone grafting (free non-vascularized half fibula) in the treatment of femoral shaft non-union of 21 patients. The full clinical and radiological union was seen in all the patients. On the contrary, in a work made by Won et al. [43], there was no difference in the bone union between a group of subtrochanteric non-unions treated with re-nailing with bone grafting and the other one treated without bone grafting. They conclude the outcome of subtrochanteric revision surgery are mainly influenced by fracture type (atypical/typical), the number of previous surgeries and the presence of varus and sagittal anterior angulation [41].

Additionally, iliac and fibular autologous bone grafting is associated with significant donor site morbidity and can result in limited graft availability [44]. Moreover, in the elderly population osteopenia and red to yellow bone marrow replacement precludes the harvesting of graft from the iliac crest. In these cases, the RIA system from the contralateral femur (as we previously described in our experience [20, 45]) represents a wise choice, together with the use of growth factors and scaffolds. This was known as the triangular concept, afterwards modified by Giannoudis et al. [9] into the “Diamond concept”, with the addition of mechanical stability to these three dimensions of biological enhancement of bone healing, highlighting the fundamental role of stability in these fractures.

The strengths of our study include the use of a single type of implant and the single-stage surgical procedure, performed by the same surgeon. The weaknesses of our study include its retrospective design, the lack of a control group and the relatively small sample size.

## Conclusions

This study shows that the treatment of inter- and sub-trochanteric non-unions with a 95° blade plate, medial strut allograft, and bone autograft obtained with RIA system, together with a varus malalignment correction, leads to a high percentage of bone union, with a low incidence of complications and good clinical outcome.

## Data Availability

The datasets used and/or analyzed during the current study are available from the corresponding author on reasonable request.

## Abbreviations

HHS: Harris Hip Score
SF-12: Short-Form 12
RIA: Reamer Irrigator Aspirator
CRP: C- Reactive Protein
ESR: Erythrocyte Sedimentation Rate
VITO: Valgus Intertrochanteric Osteotomy
VSTO: Valgus Subtrochanteric Osteotomy
LMWH: Low Molecular Weight Heparin
